# Constipation in the Pediatric Emergency Department: Clinical Presentations, Diagnostic Context and Testing Patterns

**DOI:** 10.3390/diseases14070239

**Published:** 2026-07-02

**Authors:** Julia Leszkowicz, Kinga Miaśkiewicz, Marcin Wieczorek, Magdalena Dettlaff-Dunowska, Agnieszka Szlagatys-Sidorkiewicz

**Affiliations:** 1 Department of Paediatrics, Gastroenterology, Allergology & Paediatric Nutrition, Medical University of Gdańsk, Nowe Ogrody 1/6, 80-803 Gdańsk, Poland; 2 Copernicus Hospital, Nowe Ogrody 1/6, 80-803 Gdańsk, Poland

**Keywords:** constipation, abdominal pain, emergency department, differential diagnosis, pediatric patients

## Abstract

**Background**: Constipation in children often presents with non-specific symptoms, which can complicate its recognition in the pediatric emergency department (PED). **Aim**: This study aimed to characterize the clinical presentations, diagnostic context and testing patterns of children discharged with constipation from a tertiary pediatric emergency center. **Methods**: A retrospective analysis of medical records of patients under 18 years of age was conducted for patients who presented to the PED of a tertiary hospital in northern Poland from 2021–2024 and were ultimately discharged as K59.0 ICD-10 code (constipation). Demographic data, symptoms reported upon admission, and laboratory and imaging tests performed were collected and reviewed. **Results**: PED visits discharged with ICD-10 code K59.0 accounted for 2.97% of all 34,278 PED visits during study period. Among 1017 patients discharged with ICD-10 code K59.0, only 26.5% reported constipation as their main complaint. The most common complaints were abdominal pain (61.6%), vomiting (14.4%), and urinary symptoms (4.9%). Commonly suspected initial diagnoses were urinary tract infections or acute appendicitis. More complete documentation of constipation-related symptoms showed an exploratory association with less intensive diagnostic testing. **Conclusions**: Constipation should be routinely considered in children presenting to the PED with abdominal pain, vomiting, urinary symptoms, or rectal bleeding, even when bowel problems are not the main complaint. Structured history taking supported by simple diagnostic tools could help standardize assessment and support patient selection for further testing, although prospective studies are needed to determine clinical outcomes.

## 1. Introduction

Constipation is a condition frequently affecting children worldwide, with a global prevalence of around 9.5% [1]. Functional constipation accounts for over 95% of constipation cases in healthy children aged one year and above and is especially prevalent in preschoolers [2,3]. The diagnosis of this disorder is based on Rome criteria. According to these guidelines, functional constipation is defined by the presence of at least two specific symptoms occurring weekly for a minimum of one month, with no other medical condition that could explain them. The symptoms include two or fewer defecations per week, excessive stool retention, painful or hard bowel movements, large-diameter stools, and the presence of a large fecal mass in the rectum. In toilet-trained children, fecal soiling after acquisition of toileting skills and large-diameter stools that may obstruct the toilet are also included [4]. Although Rome criteria provide a clinical framework for diagnosing functional constipation, the emergency department setting often involves incomplete symptom documentation and broader differential diagnosis context. Therefore, constipation-related visits identified retrospectively through ICD-10 discharge (K59.0) coding should not be interpreted as a homogenous population of children with prospectively confirmed functional constipation. Such cohorts may include children with functional constipation, constipation as a concomitant clinically relevant finding or mixed symptoms.

This study aimed to characterize the clinical presentations, diagnostic context and testing patterns of children discharged with constipation coded as K59.0 from a tertiary pediatric emergency center. We conducted a retrospective study of patients presenting to the largest tertiary pediatric emergency department (PED) in northern Poland, integrated with a tertiary referral center for pediatrics, gastroenterology, allergology and nutrition between 2021 and 2024.

## 2. Materials and Methods

### 2.1. Data Collection

A retrospective single-center observational study was conducted based on the hospital records of 1017 patients admitted to the pediatric emergency department of a tertiary hospital in Gdańsk, Poland. Electronic data of every patient aged 0 to 18 years who was admitted to the PED between 2021 and 2024 and who was discharged from pediatric emergency department with ICD-10 code K59.0 were analyzed. After obtaining approval from the Human Research Ethics Committee of the Medical University of Gdańsk (KB/339/2025) the data were anonymously collected via the hospital’s electronic records system and then analyzed in terms of information determined during history taking (Table S1), physical examination, and additional tests (if considered necessary). The list of tests is attached as Supplementary Materials. History taking was analyzed in terms of documented Rome-IV-related features of constipation rather than formal fulfillment of Rome IV criteria for functional constipation [3]. The data was extracted by standardized chart review using a predefined reconciliation framework. Three researchers systematically reviewed the medical records according to prespecified rules, and discrepant or ambiguous cases were resolved by consensus discussion.

Rome-IV-related features were assessed retrospectively—for each feature, we distinguished whether the symptom was recorded (asked about), and, if documented, whether this symptom was present or absent. Undocumented symptoms were not interpreted as absent. Patients were categorized according to the number of documented Rome-IV-related features present in the medical record. The term ‘at least two documented Rome-IV-related features’ was used to describe cases in which the available history was consistent with two or more constipation-related Rome IV features.

Visits were included when constipation was recorded as clinically relevant during the PED hospitalization, regardless of whether it was the main presenting complaint or part of a broader clinical presentation. Therefore, cases with comorbidities or concomitant symptoms, such as fever, upper respiratory tract infection, urinary tract infection, vomiting, abdominal pain, or rectal bleeding, were retained if constipation was documented as a relevant discharge diagnosis. This approach was intended to reflect the real-world diagnostic context of constipation in emergency care, where constipation may be the primary problem, a concomitant finding, or part of a mixed symptom complex.

### 2.2. Data Analysis

IBM SPSS Statistics for Windows, version 29.0.2 was used for the analysis of the data. The characteristics of the sample were analyzed using descriptive statistics. Categorical data were presented as numbers and percentages, and continuous data were presented as means and standard deviation or medians, depending on the distribution. The chi-square test and Fisher’s exact test were used to compare categorical variables between groups. In case of significant results, Cramér’s V coefficient was used to assess the strength of the relationship between the variables. The relationships between two quantitative variables were assessed using Pearson linear correlation coefficient. Tests were two-tailed with a confidence level set at 95%. *p*-values of less than 0.05 were considered statistically significant. All inferential analyses were considered exploratory and hypothesis generating. No formal adjustment for multiple comparisons was applied; therefore, *p*-values were interpreted as nominal and together with effect-size estimates.

## 3. Results

### 3.1. Sample Characteristics

PED visits discharged with ICD-10 code K59.0 accounted for 2.97% of all PED visits during the study period (1017 of 34,278 PED patients), with 54.2% (*n* = 551) being male. The mean age of patients was 7 years (6 years 7 months for males and 7 years 6 months for females). The most common age group was primary school children, with a slight predominance of males. Table 1 shows demographic data of the analyzed population.

Seasonal analysis revealed a higher proportion of constipation during the winter months, particularly in January, October and December (ranging from 9.6% to 10.9%), with a notable decline during the summer months from July to September (ranging from 6.6% to 7.2%).

The highest number of admissions occurred in the afternoon and early evening (2:00–8:00 p.m.), accounting for an average of 36.2% of all admissions. Late evening and nighttime (8:00 p.m.–2:00 a.m.) also had a significant share, with an average of 29.1%. There were fewer admissions between 8:00 a.m. and 2:00 p.m. (27.8%), while the lowest numbers of children were admitted to PED in the early morning (2:00–8:00 a.m.)—only 6.9% on average.

### 3.2. Causes of Admission

The most common complaint at admission was abdominal pain, which constituted 61.6% of all cases. Among children with available data, the most frequently reported location was the periumbilical area (12.5%) and the left side of the abdomen (11.7%), followed by the lower quadrants (10.9%), lumbar region (8.6%) and right side (6.5%). In 7.5% of cases, abdominal pain was reported, but its exact location was not specified. The location of abdominal pain was not documented in 39.6% of cases.

**Figure 1:**
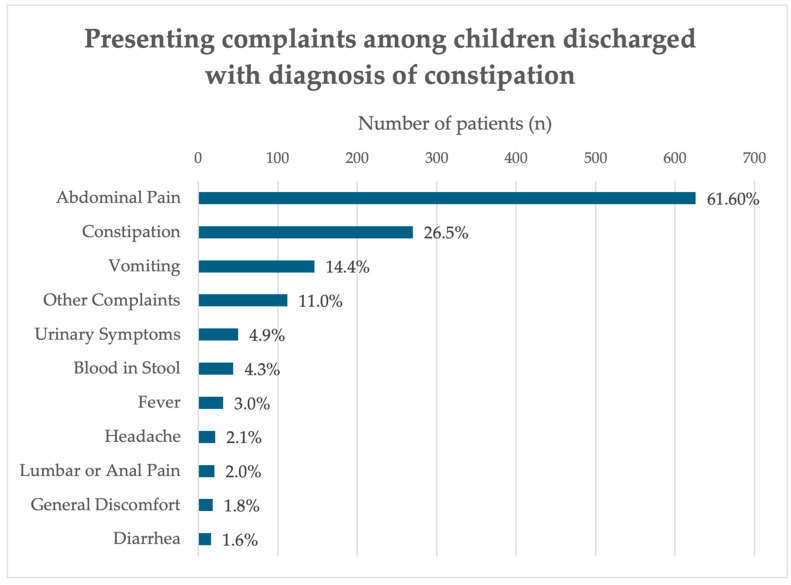
Presenting complaints among children in the Pediatric Emergency Department with final diagnosis of constipation. Percentages refer to the total number of children within a cohort.

Other common causes of admission were constipation (26.5%) and vomiting (14.4%). Less common complaints included frequent urination or urinary retention (4.9%), blood in stool (4.3%), fever (3.0%), headaches (2.1%), pain in the lower back or anal area (2.0%), anxiety or unease (1.8%) and diarrhea (1.6%). Other causes, such as injuries, check-ups, and chest pain, constituted 11.0% of reports. Figure 1 shows the causes of admissions in percentage.

Within the cohort, a documented history of constipation was present in 438/1017 children (43.1%). Previous treatment for constipation was recorded in 310/1017 cases (30.5%). The most frequently documented previous treatment were macrogol-based preparations in 198/310 children (63.9%), rectal enemas or microenemas in 76/310 (24.5%), lactulose in 52/310 (16.8%), and suppositories in 51/310 (16.5%). Less frequently recorded modalities included dietary interventions in 14/310 children (4.5%), senna-based preparations in 12/310 (3.9%), trimebutine in 8/310 (2.6%), probiotics in 5/310 (1.6%), mechanical stimulation of defecation in 4/310 (1.3%), bisacodyl in 3/310 (1.0%), sodium picosulfate in 2/310 (0.6%), and paraffin oil in 2/310 (0.6%). These categories were not mutually exclusive, as more than one treatment modality could be recorded for the same child.

### 3.3. Documented Rome-IV-Criteria-Related Features

In the initial stage of the analysis, the frequency of individual Rome IV criteria symptoms was assessed in two age groups: children under four years of age and children aged four years and over, according to division proposed in Rome IV criteria. This descriptive, qualitative analysis outlines differences in constipation symptom profiles by age, providing context for subsequent analyses of the diagnostic process and clinical decisions.

Based on available documentation, 287 of 1017 children (28.2%) had records consistent with at least two Rome-IV-related features of constipation. In 729 children (71.7%), fewer than two Rome-IV-related features were documented. Rome-IV-criteria-related variables were heterogeneous within the cohort: symptom duration was available in 100% of records, stool frequency in 67.6%, painful defecation in 25.1%, fecal soiling in 20.0%, large stools or toilet obstruction in 13.1%, and stool withholding behavior in 6.4%. Because of the retrospective design, children with fewer than two documented features should not be interpreted as definitively not fulfilling Rome IV criteria—it rather demonstrates limited or incomplete documentation of constipation-specific history in the PED record. This likely reflects routine emergency department documentation practice, where once constipation was considered sufficiently supported clinically, further detailed recording of additional Rome IV criteria symptoms was often not pursued.

**Figure 2:**
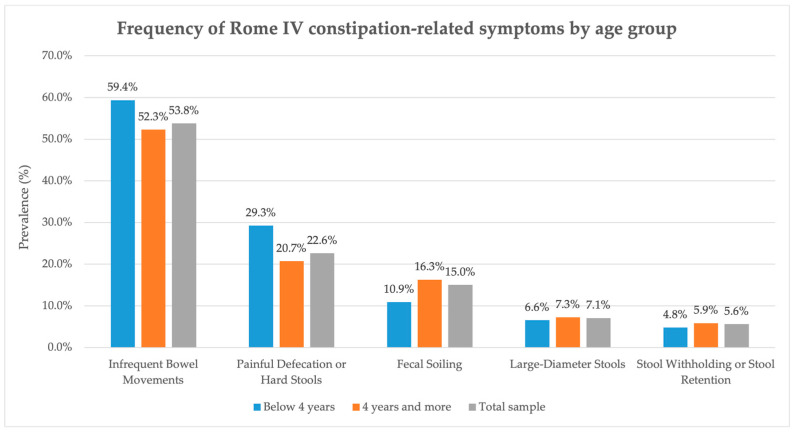
Frequency of Rome IV criteria symptoms in children under and over 4 years of age. Percentages refer to the number of children in each age group.

Of all the Rome IV symptoms analyzed (as shown in Figure 2), infrequent bowel movements were the most frequently reported feature. They were more common in children under four years of age than in older children, however, the effect size was small (*p* = 0.001, V = 0.12). Painful or hard bowel movements were numerically more frequent in younger children, but this difference was not statistically significant (*p* = 0.083, V = 0.11). Fecal soiling showed no meaningful difference between age groups (*p* = 0.914, V = 0.01). Likewise, stool withholding was reported at comparable frequencies across age groups (Fisher’s exact *p* = 0.684, V = 0.07). Large stool volume also did not differ significantly by age group (*p* = 0.526, V = 0.10). However, this result should be interpreted cautiously because some expected frequencies in contingency table cells were below 5, and one response category appeared to contain an isolated miscoded value.

### 3.4. Suspected Diagnoses

**Figure 3:**
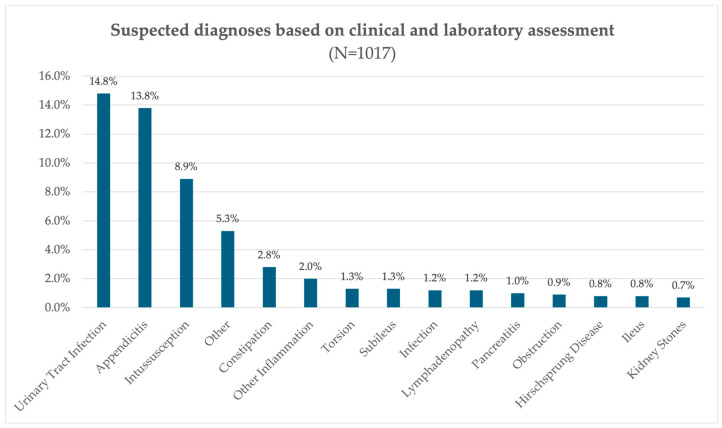
Suspected diagnoses based on history, physical examination and laboratory tests. Values are presented as percentages of patients in whom a given diagnosis was suspected. Percentages refer to the total number of children within a cohort.

Based on analyzed records, physical examination and subsequently referred tests, clinicians most often suspected urinary tract infection (14.8%), followed closely by acute appendicitis (13.8%) and intussusception (8.9%), along with other surgical conditions, such as torsion (1.3%), subileus (1.3%), ileus (0.8%), or other kind of obstruction (as shown in Figure 3). Constipation was considered in 2.8% of patients.

The next stage of the analysis examined the co-occurrence of Rome IV constipation criteria with specific suspected diagnoses, separately for children under 4 years of age and for those aged 4 years and over. These exploratory analyses were based on correlation coefficients and were intended to describe patterns of co-occurrence. In line with the predefined interpretative criterion, only correlations with an absolute value of ≥0.30 were considered further as these were of moderate strength and potentially clinically meaningful. Correlations below this threshold were regarded as weak and not interpreted.

In younger children, exploratory correlation analyses showed several moderate associations between documented Rome-IV-related features and suspected diagnoses. The strongest positive correlations were found between large stool volume and constipation (r = 0.63). In addition, soiling showed a moderate positive correlation with intussusception (r = 0.63). Moderate negative correlations were observed between infrequent bowel movements and acute appendicitis (r = −0.33), as well as between large stool volume and Hirschsprung disease (r = −0.50), suggesting that these symptoms were less commonly documented in association with these suspected diagnoses in this subgroup.

Correlation analyses examining the relationship between reported constipation (as a symptom) and the main suspected diagnoses revealed no significant association, only two exceptions were observed. Reported constipation was not significantly associated with urinary tract infections (r = 0.01, *p* = 0.887), inflammation (r = −0.04, *p* = 0.482), lymphadenopathy (r = 0.01, *p* = 0.915), infection ( r = −0.01, *p* = 0.915), intestinal obstruction (r = 0.08, *p* = 0.138), acute pancreatitis (r = −0.07, *p* = 0.170), kidney stones (r = −0.06, *p* = 0.253), organ torsion (r = −0.04, *p* = 0.427) or any other suspected diagnosis. Reported constipation showed weak negative correlation with suspected acute appendicitis (*r* = −0.21, *p* < 0.001) and intussusception (*r* = −0.11, *p* = 0.041), suggesting that these diagnoses were less likely to be made when constipation was reported. Conversely, there was a moderate positive correlation between reported constipation and suspected Hirschsprung’s disease (*r* = 0.34, *p* < 0.001), although this finding should be interpreted cautiously because it likely reflects targeted clinical suspicion in a small subgroup rather than a generalizable diagnostic pattern.

### 3.5. Abdominal Pain—Predominant Presentation

As abdominal pain is the leading symptom in this cohort, it was thoroughly assessed. To compare the frequency of reasons for admission to the pediatric emergency department, and suspected diagnoses between patients with acute and chronic abdominal pain (less vs. more than 60 days [5]), Fisher’s exact tests were performed. The results are reported in Table 2.

A comparison of reasons for presentation to the pediatric emergency department between patients with acute and chronic abdominal pain revealed a significant difference only in cases of constipation. Constipation was reported more frequently as the reason for presentation in patients with chronic abdominal pain than in those with acute abdominal pain (45.1% vs. 24.4%; Fisher’s exact test, *p* = 0.002; Cramér’s V = 0.108).

### 3.6. Diagnostic Testing and Clinical Assessment

**Figure 4:**
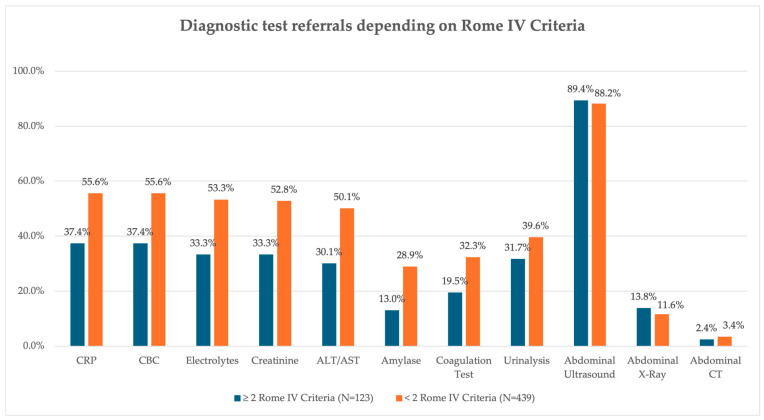
Diagnostic tests performed among children who underwent at least one laboratory or imaging investigation, stratified according to documented Rome-IV-related features. Percentages indicate the proportion of investigated patients within each group who underwent a given test. Groups were defined as children with documentation consistent with at least two Rome-IV-related features (*n* = 123) and children with fewer than two documented features (*n* = 439). CRP—C-reactive protein, CBC—complete blood count, ALT—alanine aminotransferase, AST—aspartate aminotransferase, CT—computed tomography.

Diagnostic testing was analyzed according to the number of documented Rome-IV-related features. Among children who underwent at least one laboratory or imaging investigation (*n* = 562), patients were stratified into two groups: children with documentation consistent with at least two Rome-IV-related features (*n* = 123) and children with fewer than two documented features (*n* = 439). Percentages in Figure 4 indicate the proportion of patients within each group who underwent a given diagnostic test, children without a given test were counted as not tested.

Within this investigated subgroup (*n* = 562), any laboratory testing was more frequent among children with fewer than two documented Rome-IV-related features than among those with documentation consistent with at least two features (276/439, 62.9% vs. 59/123, 48.0%; χ^2^ = 8.863, *p* = 0.003, Phi = 0.13). This pattern was observed for CRP (244/439, 55.6% vs. 46/123, 37.4%; *p* < 0.001, Phi = 0.15), complete blood count (243/439, 55.4% vs. 46/123, 37.4%; *p* < 0.001, Phi = 0.15), electrolytes (234/439, 53.3% vs. 41/123, 33.3%; *p* < 0.001, Phi = 0.17), renal parameters (232/439, 52.8% vs. 41/123, 33.3%; *p* < 0.001, Phi = 0.16), liver parameters (220/439, 50.1% vs. 37/123, 30.1%; *p* < 0.001, Phi = 0.17), pancreatic parameters (127/439, 28.9% vs. 16/123, 13.0%; *p* < 0.001, Phi = 0.15), and coagulation tests (142/439, 32.3% vs. 24/123, 19.5%; *p* = 0.006, Phi = 0.12). In contrast, urinalysis did not differ significantly between groups (174/439, 39.6% vs. 39/123, 31.7%; *p* = 0.109, Phi = 0.07). Imaging studies were performed at comparable frequencies in both groups, including abdominal ultrasound (387/439, 88.2% vs. 110/123, 89.4%; *p* = 0.696, Phi = 0.02), abdominal X-ray (51/439, 11.6% vs. 17/123, 13.8%; *p* = 0.508, Phi = 0.03), and abdominal CT (15/439, 3.4% vs. 3/123, 2.4%; Fisher’s exact test, *p* = 0.775, Phi = 0.02).

Digital rectal examination was documented in 472 of 1,017 patients (46.4%); it was not documented in 545 patients (53.6%), in two cases (0.2%) the record was insufficient or ambiguous. The performance of DRE was not associated with initial clinical suspicion of constipation (13/472, 2.8% vs. 15/543, 2.8%; χ^2^ = 0.001, *p* = 0.977, Phi = 0.001). Among children who underwent DRE, the most frequently documented finding was fecal masses or stool in the rectal ampulla (355/472, 75.2%). Other findings included a widened rectal ampulla (83/472, 17.6%), anorectal fissure or mucosal/skin lesions (44/472, 9.3%), an empty rectal ampulla or no fecal retention (30/472, 6.4%), fecal soiling of the perianal area (28/472, 5.9%), altered sphincter tone (9/472, 1.9%), and rectal or mucosal prolapse (2/472, 0.4%). These categories were not mutually exclusive.

Palpable fecal masses were documented in 602 of 1010 patients with available data (59.6%). They were more frequent among children with documentation consistent with at least two other Rome-IV-related features than among those with fewer documented features (218/285, 76.5% vs. 384/725, 53.0%; χ^2^ = 47.025, *p* < 0.001, Phi = 0.22). This association was moderate in magnitude and reflects the relationship between physical examination findings and more complete constipation-specific documentation, rather than the effect of DRE itself on diagnostic recognition.

Moreover, in 8.6% of cases, constipation was recorded as a secondary diagnosis, although the available documentation did not contain sufficient Rome-IV-related features to retrospectively confirm the diagnosis. In children with a prior history of constipation, the percentage of laboratory tests ordered did not differ significantly between children under the care of a gastroenterologist and the others (29.3% vs. 32.8%; *p* = 0.51; OR 0.85, 95% CI 0.53–1.36).

### 3.7. Final Diagnosis

Out of 1017 patients, pediatric surgical consultation was performed in 22 patients, gynecological consultation in six patients, and orthopedic consultation in one patient. In these cases, acute surgical, gynecological, or orthopedic causes of symptoms were excluded before constipation was verified as the final diagnosis. When clinically indicated, PED physicians could also consult a pediatric gastroenterology fellow from the affiliated gastroenterology ward. Enema was administered in 17.9% of cases. In cases of presence of red flags or insufficient symptom improvement after initial treatment, 9.8% were transferred to the gastroenterology ward immediately, and 3.4% were electively scheduled. Among the subset of 134 patients referred to our pediatric gastroenterology ward (due to presence of red flags or unsatisfactory initial treatment at the PED), functional constipation as the sole final diagnosis was documented in 70 cases, whereas 55 patients received additional concomitant diagnoses. A different final diagnosis was established in nine patients—as shown in Table S2. Regardless of the presenting symptoms, all patients were discharged with a recommendation for follow-up and ongoing care under a general practitioner or pediatric gastroenterologist to exclude potential organic causes of constipation.

## 4. Discussion

### 4.1. Principal Findings

This retrospective analysis indicates that constipation in the pediatric emergency department is frequently identified in the context of non-specific abdominal, urinary, or gastrointestinal symptoms, rather than as a straightforward presenting complaint. Globally, the prevalence of constipation among pediatric patients presenting to emergency departments ranges widely from 0.7% to 30%, depending on the study design, population, and diagnostic criteria used [6]. The prevalence of constipation among pediatric emergency department patients in Poland is not reported in recent European systematic reviews or meta-analyses. At our department, which serves as a referral center for surgical and gastroenterological cases for children from northern Poland (population of Gdańsk is almost 2.4 mln [7]), constipation-coded (K59.0) visits accounted for almost 3%, which is slightly higher than the 0.4–2.1% range reported from Italy and Canada [8,9] for overall PED visits. This higher proportion may reflect differences in study design, healthcare systems, patient populations and ICD coding practices. Nonetheless, just like in other centers, despite well-defined diagnostic criteria for constipation, additional diagnostic testing remained common in clinical practice, mostly due to probable fear of missing an acute condition [10,11,12,13]. Constipation was rarely reported as a main symptom—much more often the patients reported abdominal pain, vomiting, urinary symptoms or blood in the stool. For this reason, excluding acute states was necessary. Results show that documentation of Rome IV criteria symptoms was oftentimes incomplete, which demonstrates the difference between ideal diagnostic criteria and real practice.

### 4.2. Abdominal Pain—A Main Symptom

The most frequently reported symptom was abdominal pain. Generally, abdominal pain is one of the most common symptoms in children and adolescents and is estimated to account for approximately 5% of unscheduled office visits [14]. While compatible with a diagnosis of constipation, abdominal pain may also indicate other pathologies and thus require careful differential assessment. Findings reported by other emergency departments show that, among children with abdominal pain, 11.3% had a discharge diagnosis of constipation and 45.5% underwent diagnostic imaging [15].

In our analysis, the pain location was heterogeneous among children with documented localization, which may reflect the broad differential diagnosis considered in PED settings. However, pain location was not documented in nearly 40% of patients reporting abdominal pain, therefore these findings should be interpreted cautiously and regarded as descriptive. The incomplete documentation may indicate an area where history taking could be more consistently structured, although this may be challenging in younger children who cannot reliably localize pain [16]. In addition, caregivers or patients may provide incomplete histories, particularly for symptoms perceived as embarrassing, such as withholding or soiling [15], or they may not recognize the symptoms properly [17,18].

The higher frequency of constipation as a presenting complaint among children with chronic abdominal pain suggests that caregivers and clinicians may be more likely to recognize constipation when symptoms persist over time. In contrast, acute abdominal pain more often triggers evaluation for urgent surgical or infectious conditions, which may partly explain broader diagnostic testing in this group.

### 4.3. Clinical Presentations of Constipation Mimicking Acute Conditions

Aside from abdominal pain, constipation may present with symptoms that overlap with acute surgical, urological, infectious or other conditions. We aimed to describe symptom patterns as they were documented in routine pediatric emergency care.

The largest cluster involved surgical conditions. One of the most common causes of referral to the PED by general practitioners was suspicion of appendicitis, involving symptoms such as abdominal pain poorly reacting to painkillers, abdominal distention, positive Blumberg sign, sometimes low-grade fever, and inability to pass stool for a few days [19]. For diagnosis of appendicitis, imaging of the appendix upon abdominal ultrasound is a first-line modality [20], which may also demonstrate stool retention in selected cases [21].

Intussusception, subileus or ileus were suspected when a patient presented with vomiting, abdominal distension and lazy bowel sounds that might suggest GI tract occlusion [22]. Abdominal radiography may be considered when bowel obstruction is clinically suspected, depending on the overall clinical picture [3,23].

A part of admissions involved lower GI tract bleeding—most patients presented with minor fresh-blood bleeding, indicating anal fissure, a result of passing hard stools. Rectal examination, although used carefully, is often indicative of diagnosis [24]. Only a small number of children merited admission to the hospital or referral for rectosigmoidoscopy, suggesting that a majority of visits involved non-life-threatening bleeding [25].

Another presentation that may overlap with constipation involves urological symptoms (i.e., urinary tract infection, urinary retention, nephrolithiasis). Those incidents mostly covered dysuria and inability to urinate, with or without fever. If examination was not sufficient for diagnosis, urine analysis was referred for exclusion/confirmation of UTI [26]. Some patients reported acute urinary retention (due to obstruction) [27]—in those cases, the symptoms appeared to improve after disimpaction with enema. According to the literature, along with balanoposthitis, constipation is one of the most common causes of acute urinary retention in children [28], thus should be considered in differential diagnosis.

Another pattern involved symptoms suggestive of infection. Patients were presenting with nausea and vomiting or diarrhea, at times with accompanying fever (if overlapped with GI tract infection). Dysmotility of the upper GI tract could be explained by retention and obstruction in the lower tract [29]. In the case of diarrhea, a common pathophysiological cascade leading to soiling in children begins with stool withholding, often due to fear of painful defecation, which results in progressive fecal impaction and subsequent overflow incontinence, as liquid stool leaks around the impacted mass without the child’s voluntary control [2].

These patterns emphasize that constipation in the PED is often recognized within a broader diagnostic context rather than through a single typical symptom. The concept of mimicking presentations should not reduce diagnostic vigilance.

### 4.4. Diagnostic Tests and Clinical Uncertainty

Diagnostic testing in this cohort was largely shaped by clinical uncertainty and the need to exclude acute conditions that may present similarly to constipation. Abdominal ultrasound and CRP were routinely performed even if history supported the diagnosis of constipation. In our cohort, among children who underwent diagnostic testing, less complete Rome-IV-related documentation was associated mainly with more frequent laboratory testing, whereas imaging use was similar between groups. Comparable patterns of testing have been reported in other institutions, suggesting that similar diagnostic challenges may occur in other PED settings. Gatto et al. report that 45.5% of PED patients with abdominal pain underwent one or more imaging studies. Specifically, abdominal X-rays were performed in 36.4% patients, abdominal ultrasound in 20.8%, and blood tests were conducted in 21.4% of patients [15]. Abdominal radiographs were used frequently in the ED diagnosis and management of constipation, particularly in older children and those with abdominal pain and emesis and were associated with increased length of stay [30].

Digital rectal examination was documented in nearly half of the cohort and, when performed, most frequently revealed fecal masses or stool in the rectal ampulla. However, DRE as a clinical action should be distinguished from its findings. Because all included patients had a final discharge diagnosis of constipation, this study could not assess whether DRE was associated with final diagnostic confirmation. DRE findings may support clinical assessment in selected patients, particularly when constipation-specific history is incomplete, the diagnosis remains uncertain, or an anorectal or organic condition needs to be excluded.

Although DRE is not required for the diagnosis of constipation in all patients, it may be useful in selected cases. Rome IV criteria indicate that, if only one criterion is present and the diagnosis is uncertain, a digital examination of the anorectum is recommended to confirm the diagnosis and exclude underlying medical conditions [4]. Furthermore, rectal exam could be useful in cases of occult constipation (when patients do not report problems with defecation, but rather recurrent abdominal pain) [31,32].

Another issue is the fact that studies show overdiagnosis of constipation mainly based on solely radiographic diagnosis of fecal retention following inappropriate abdominal X-ray request [33]. In our study, there was also a group (nearly 9%) of patients receiving diagnosis of constipation despite incomplete documentation of constipation-specific symptoms. Those patients were usually suspected of ingestion of a foreign body, and X-rays revealed fecal retention, so the patients were given K59.0 diagnoses along with macrogols to expel the foreign body more easily. Previous studies suggest that a targeted clinical examination, including DRE in selected cases, may reduce reliance on abdominal imaging [34].

The supplementary follow-up data further support the diagnostic heterogeneity of constipation-coded PED visits. Among children referred to the pediatric gastroenterology ward, functional constipation alone was confirmed in about half of the subgroup, while the other half received alternative or concomitant diagnoses. Importantly, this referred subgroup should not be interpreted as representative of all constipation-coded PED visits, because referral was more likely in children with red flags, persistent symptoms, or diagnostic uncertainty. Nevertheless, these data emphasize the need for careful follow-up, especially in children with atypical presentations or incomplete response to initial treatment.

### 4.5. Clinical Implications for PED

Our findings suggest that pediatric emergency departments may benefit from practical, setting-specific guidance and targeted education on the recognition and assessment of constipation in children. The present study was designed and analyzed using Rome-IV-based symptom domains. Although Rome V criteria became available during revision [35], they were not used for data extraction or analysis and should be considered only as context for future research. Rome V provides a more structured framework for pediatric disorders of gut–brain interaction, including clearer organization by anatomical region. This may make symptom-based criteria easier to apply in emergency department settings, where long-term clinical information is often limited.

Children with at least two documented Rome-IV-related features were less likely to undergo additional diagnostic testing than those with fewer documented features. This may suggest that diagnostic decisions in the ED are influenced by alarm symptoms, clinical uncertainty or the physician’s experience rather than by strict application of symptom-based criteria alone. To achieve an accurate diagnosis, recognition of constipation in the ED setting may benefit from more structured symptom assessment, as research shows that knowledge among primary care pediatricians (who refer patients to PEDs) regarding constipation is inadequate [10]. There are significant discrepancies not only between specialists but also in diagnostic and treatment practices [36,37,38,39].

Using simple checklists and aids, such as Rome criteria, Bristol Stool Scale [40], Infant Stool Form Scale [41] or a red flags list, for constipation may help standardize the diagnostic pathway and support patient management, although their impact should be evaluated prospectively. The most important point should be creating a simple, systematic and routine approach to constipation in the PED. Thus, the aim should not be to omit diagnostic testing, but rather to improve patient selection and ensure that investigation is guided by clinical presentation, alarm features, and the likelihood of identifying clinically relevant pathology. Importantly, the initial emergency department assessment should not replace appropriate follow-up, particularly in children with atypical symptoms, alarm features, or incomplete response to treatment.

### 4.6. Limitations

This study has several key limitations that should be considered when interpreting the results. Given its retrospective observational design, the study is subject to documentation-related limitations and potential bias, and its findings should be interpreted as descriptive rather than causal. In addition, multiple exploratory comparisons and correlation analyses were performed without formal adjustment for multiplicity. Therefore, statistically significant findings should be interpreted cautiously, particularly when effect sizes were small or subgroup counts were limited. At the same time, it reflects routine clinical practice and offers clinically relevant real-world observations from a tertiary pediatric emergency setting. Secondly, although the sample size is large, the findings may not be applicable to other institutions, regions, or healthcare systems with different patient populations or management approaches.

Additionally, this center, due to the nature of the pediatric gastroenterology ward where most physicians work, introduces a bias. As a tertiary center (the only one with pediatric gastroenterology in the voivodship), local referral patterns and clinician experience might have influenced diagnostics, because physicians working in the PED are 1st to 5th year pediatrics residents undergoing training at the gastroenterology ward. Including data from other hospitals, especially those focusing on general pediatrics, would be beneficial.

Case identification relied solely on the ICD-10 code K59.0 (functional constipation). This method may have missed relevant cases that were miscoded or diagnosed under different, non-specific codes (such as abdominal pain—R10.4, nausea and vomiting—R11, fecal soiling—R15.0 or hematemesis—K92.0), potentially causing misclassification bias. The retrospective pediatric emergency department design also did not allow a reliable distinction between constipation and irritable bowel syndrome with constipation (IBS-C). This distinction requires longitudinal assessment of abdominal pain, its relationship with defecation and stool pattern, and persistence or resolution of pain after constipation treatment. These data were not systematically available in our cohort; therefore, abdominal pain was interpreted as a presenting symptom rather than evidence of IBS-C. We cannot exclude that, in some children initially discharged with constipation (K59.0), the diagnosis may have been reconsidered during follow-up or specialist evaluation.

Lastly, the study did not include immediate treatment or analysis of follow-up after the initial emergency department visit. Therefore, it was not possible to assess long-term outcomes, recurrence rates, or whether patients were subsequently diagnosed with other gastrointestinal conditions.

## 5. Conclusions

This retrospective study highlights the heterogeneous presentation of constipation in the pediatric emergency department and the frequent use of additional diagnostic testing in this population. The findings suggest that more systematic documentation of constipation-related symptoms and alarm features may be associated with clearer diagnostic assessment and could help support decisions regarding further testing. The observations support considering constipation in differential diagnosis of children presenting to the PED with abdominal pain, vomiting, urinary tract symptoms and rectal bleeding, even if bowel movement problems were not reported as a main complaint.

However, because of the retrospective observational design, these results should be interpreted as descriptive and exploratory. The study cannot determine whether structured history taking or simple clinical tools directly improve diagnostic recognition, reduce unnecessary investigations, or enhance patient safety. Prospective studies are needed to evaluate whether such approaches can improve clinical decision making and management outcomes in emergency settings.

## Figures and Tables

**Table 1:** Baseline characteristics of described cohort—children discharged from pediatric emergency department with diagnosis of constipation.

Variable	*n* = 1017
Age, years (mean ± SD)	7.07 ± 4.47
Median age, years (IQR)	6.0 (3.5–10)
Age range, days–years	16 days–18 years
Sex, male (%)	551 (54.2%)
Sex, female (%)	466 (45.8%)
Children > 4 years, *n* (%)	740 (72.8%)
Children < 4 years, *n* (%)	277 (27.2%)

SD = standard deviation, IQR = interquartile range.

**Table 2:** Causes of admission to the Pediatric Emergency Department in patients with acute and chronic abdominal pain.

	Acute Abdominal Pain		Chronic Abdominal Pain		
	*n*	%	*n*	%	*p*
Causes of admission to the PED					
Abdominal pain	556	62.9	31	60.8	0.767
Constipation	216	24.4	23	45.1	0.002
Vomiting	131	14.8	4	7.8	0.219
Other	94	10.6	4	7.8	0.644
Urinary retention/pollakiuria	41	4.6	3	5.9	0.728
Blood in stool	39	4.4	3	5.9	0.495
Fever	28	3.2	1	2.0	>0.99
Headache	21	2.4	0	0.0	0.623
Lumbar/anal pain	17	1.9	1	2.0	>0.99
Unease	16	1.8	0	0.0	>0.99
Diarrhea	15	1.7	0	0.0	>0.99

## Data Availability

The data that support the findings of this study are available from Julia Leszkowicz upon e-mail request.

## Supplementary Materials

The following supporting information can be downloaded at: https://www.mdpi.com/article/10.3390/diseases14070239/s1, Table S1: Information collected during the analysis; Table S2: Final diagnoses (concomitant diseases and different conditions) posed in subgroup of patients admitted to the department of pediatric gastroenterology.

## Abbreviations

The following abbreviations are used in this manuscript:

PED	Pediatric Emergency Department
ESPGHAN	European Society for Paediatric Gastroenterology, Hepatology and Nutrition
NASPGHAN	North American Society for Pediatric Gastroenterology, Hepatology and Nutrition
LDH	Lactate Dehydrogenase
CT	Computed Tomography
CRP	C-Reactive Protein
CBC	Complete Blood Count
GI	Gastrointestinal Tract
DRE	Digital Rectal Examination
ALT	Alanine Aminotransferase
AST	Aspartate Aminotransferase
IBS-C	Irritable Bowel Syndrome with Constipation
