# Supplementary material for: Constipation in the Pediatric Emergency Department: Clinical Presentations, Diagnostic Context and Testing Patterns
Source: Diseases. 2026 Jul 2;14(7):239. doi: 10.3390/diseases14070239 (PMC13409387; doi:10.3390/diseases14070239)
Supplement: Supplementary file 1 [file diseases-14-00239-s001.zip › diseases-4346092-supplementary.pdf]

Supplementary Materials

Table S1. Information collected during the analysis.

**Analyzed variables**	
**General data**	• Date and time of admission<br>• Age<br>• Sex
**History, as provided by the patient/parent**	• Main cause of admission<br>• Location of abdominal pain (if present)<br>• Duration of symptoms (days)<br>• History of constipation (if present) and prior treatment<br>• Care of pediatric gastroenterologist (if present)<br>• Presence of fever<br>• Diet – high/low fibre<br>• Fluid intake in liters<br>• Meeting Rome IV Criteria:<br>• Defecation incidence <2 per week<br>• Stool texture - hard/solid<br>• Soiling – if present<br>• Defecation restraining retentive posturing or excessive volitional stool retention – if present<br>• Painful or hard bowel movements – if present<br>• Large diameter stools which can obstruct the toilet – if present
**Physical examination**	• <u>Digital rectal examination and result</u><br>• Palpable stool masses in the abdomen
**Laboratory tests – if requested**	• <u>CRP</u><br>• <u>Morphology</u><br>• <u>Electrolytes</u><br>• <u>Kidney parameters</u><br>• <u>Liver parameters</u><br>• <u>Pancreas parameters</u><br>• <u>Coagulology</u><br>• <u>Urine test</u><br>• <u>Other, if performed (i.e. urine culture, B-HCG, LDH, urea)</u>
**Diagnostic imagining**	• <u>Abdominal ultrasound</u><br>• <u>Abdominal X-ray</u><br>• <u>Abdominal CT</u>

- * Highlited measured if necessary

Table S2. Final diagnoses (concomitant diseases and different conditions) posed in subgroup of patients admitted to the department of pediatric gastroenterology.

Concomitant Diseases apart from Constipation	***n***
E55 - Vitamin D deficiency	*8*
R63.3 - Feeding difficulties and mismanagement	*4*
Q43.1 - Hirschsprung's disease	*4*
K90 - Intestinal malabsorption	*4*
F84 - Pervasive developmental disorders	*3*
K52.2 - Allergic and dietetic gastroenteritis and colitis	*3*
N39.9 - Disorder of urinary system, unspecified (UTI)	*3*
A09 - Diarrhoea and gastroenteritis of presumed infectious origin	*2*
F32.8 - Other depressive episodes	*2*
G80.9 - Cerebral palsy, unspecified	*2*
J15.9 - Bacterial pneumonia, unspecified	*2*
A08 - Viral and other specified intestinal infections	*1*
E10.8 - Type 1 diabetes mellitus with other complications	*1*
J06 - Acute upper respiratory infections of multiple and unspecified sites	*1*
K21.9 - Gastro-oesophageal reflux disease without oesophagitis	*1*
K62.2 - Anal prolapse (prolapse of anus)	*1*
K62.5 - Hemorrhage of anus and rectum	*1*
K85 - Acute pancreatitis	*1*
L50 - Urticaria	*1*
N10 - Acute tubulo-interstitial nephritis	*1*
N20.1 - Calculus of ureter	*1*
N47 - Redundant prepuce, phimosis and paraphimosis	*1*
N47 - Redundant prepuce, phimosis and paraphimosis	*1*
Q79.6 - Ehlers-Danlos syndrome	*1*
Q93.9 - Monosomy and deletion from autosomes, unspecified (5q deletion)	*1*
R11 - Nausea and vomiting	*1*
R15 - Faecal incontinence	*1*
R32 - Unspecified urinary incontinence	*1*
R62.9 - Lack of expected normal physiological development, unspecified	*1*
Total	***55***

Final Diagnoses not being Constipation	***n***
A08 - Viral and other specified intestinal infections	*2*
A09 - Diarrhoea and gastroenteritis of presumed infectious origin	*2*
K51.9 - Ulcerative colitis, unspecified	*1*
K52.9 - Noninfective gastroenteritis and colitis, unspecified	*1*
K62.5 - Hemorrhage of anus and rectum	*1*
N83.5 - Torsion of ovary, ovarian pedicle and fallopian tube	*1*

R63.3 + G93.4 - Feeding difficulties and mismanagement + Encephalopathy, unspecified	*1*
Total	***9***
